# Genome-Wide Characterization of Light-Regulated Gene Expression in *Botrytis cinerea* Reveals Underlying Complex Photobiology

**DOI:** 10.3390/ijms24108705

**Published:** 2023-05-13

**Authors:** Gabriel Pérez-Lara, Consuelo Olivares-Yañez, Harm van Bakel, Luis F. Larrondo, Paulo Canessa

**Affiliations:** 1 Centro de Biotecnologia Vegetal, Facultad de Ciencias de la Vida, Universidad Andres Bello, Santiago 8370186, Chile; 2 ANID–Millennium Science Initiative–Millennium Institute for Integrative Biology (iBIO), Santiago 7500565, Chile; 3 Department of Genetics and Genomic Sciences, Icahn School of Medicine at Mount Sinai, One Gustave L. Levy Place, New York, NY 10029, USA; 4 Department of Microbiology, Icahn School of Medicine at Mount Sinai, One Gustave L. Levy Place, New York, NY 10029, USA; 5 Icahn Genomics Institute, Icahn School of Medicine at Mount Sinai, One Gustave L. Levy Place, New York, NY 10029, USA; 6 Departamento de Genetica Molecular y Microbiologia, Facultad de Ciencias Biologicas, Pontificia Universidad Catolica de Chile, Santiago 8331150, Chile

**Keywords:** *Botrytis cinerea*, light receptor, photoreceptor, phytopathogen, transcriptomics, white collar

## Abstract

*Botrytis cinerea* is a necrotrophic fungus characterized mainly by its wide host range of infected plants. The deletion of the *white-collar-1* gene (*bcwcl1*), which encodes for a blue-light receptor/transcription factor, causes a decrease in virulence, particularly when assays are conducted in the presence of light or photocycles. However, despite ample characterization, the extent of the light-modulated transcriptional responses regulated by BcWCL1 remains unknown. In this study, pathogen and pathogen:host RNA-seq analyses, conducted during non-infective in vitro plate growth and when infecting *Arabidopsis thaliana* leaves, respectively, informed on the global gene expression patterns after a 60 min light pulse on the wild-type B05.10 or ∆*bcwcl1 B. cinerea* strains. The results revealed a complex fungal photobiology, where the mutant did not react to the light pulse during its interaction with the plant. Indeed, when infecting *Arabidopsis*, no photoreceptor-encoding genes were upregulated upon the light pulse in the ∆*bcwcl1* mutant. Differentially expressed genes (DEGs) in *B. cinerea* under non-infecting conditions were predominantly related to decreased energy production in response to the light pulse. In contrast, DEGs during infection significantly differ in the B05.10 strain and the ∆*bcwcl1* mutant. Upon illumination at 24 h post-infection in planta, a decrease in the *B. cinerea* virulence-associated transcripts was observed. Accordingly, after a light pulse, biological functions associated with plant defense appear enriched among light-repressed genes in fungus-infected plants. Taken together, our results show the main transcriptomic differences between wild-type *B. cinerea* B05.10 and ∆*bcwcl1* after a 60 min light pulse when growing saprophytically on a Petri dish and necrotrophically over *A. thaliana*.

## 1. Introduction

*Botrytis cinerea* is a necrotrophic phytopathogenic fungus with a broad host range. This fungus can infect close to 1000 plants, at least half of which are economically relevant crops such as grapes and tomatoes [1]. In this context, monetary losses exceeding EUR 1 billion per year worldwide are attributed to *B. cinerea* infection [2]. Owing to its wide range of hosts and targeting several plant tissues, this fungus has been used as a model to evaluate the necrotrophic mechanisms of infection. Indeed, this necrotrophic fungus has been extensively researched, leading to significant progress in various fields such as genetics, diversity in natural populations, developmental processes, epidemiology, and agricultural practices aimed at biologically and chemically controlling it. Other research areas include signal transduction pathways, secondary metabolites, and the characterization of several molecular infection strategies. Several reviews covering these topics are available [3,4,5].

Recent advances in the understanding of *B. cinerea* biology and infection abilities have shifted from previously described infection strategies, when numerous degrading enzymes and toxins with a rather indiscriminate action on plants were characterized. Indeed, current studies focus on elucidating the different dynamics employed by the fungus when infecting plants. Accordingly, the emerging view of the *B. cinerea* virulence strategy suggests a multilayered infection process in which a collective number of plant cell death-inducing proteins [6] and other virulence factors, including those mentioned above, proceed in an ordered, regulated, and additive fashion that results in rooting plant material [1,7]. 

*B. cinerea*-plant interaction begins mainly with the arrival of conidia that germinate on the plant surface. After this first inter-organismal contact, three main stages characterize the temporal dynamics of *B. cinerea* infection, which have been observed through sophisticated image-processing and visualization tools [8]. According to the latest model detailing the temporal sequence of the infection cycle, the initial phase (occurring within 0–36 h post-infection; hpi) is characterized by several events requiring the expression of various genes such as those encoding proteins that trigger cell death. Subsequently, a more restricted intermediate phase of infection (36–48 hpi) ensues, during which, both fungal and plant cell death is observed, followed by a final stage of infection (from 48 hpi onwards) characterized by a substantial increase in fungal growth and notable plant tissue death [7]. In agreement with a series of stepwise events and following an ordered and regulated disease development, the *B. cinerea*-plant interaction process varies with the time of the day and, importantly, with lighting conditions. Thus, *B. cinerea* possesses a functional circadian clock, molecular machinery that controls time-sensitive functions throughout the day. Different lines of evidence indicate that the fungus displays its maximum pathogenic potential during nighttime [9,10,11], in the absence of light. Expectedly, *B. cinerea* can sense different light wavelengths because of the 11 photoreceptors encoded by its genome. Consequently, light induces transcriptional changes that explain light-dependent developmental functions [12] and other processes including protection against excessive light and virulence. For instance, under a 12 h:12 h light:dark (LD) photoperiod, the B05.10 wild-type strain produced macroconidiation and, importantly, reduced necrotic lesions in *Arabidopsis thaliana* and *Phaseolus vulgaris* leaves compared to constant darkness (DD) conditions. Some of these responses depend on BcWCL1, a molecularly characterized photoreceptor (PhR) and a transcription factor (TF) in *B. cinerea* [13,14,15]. 

BcWCL1 is the ortholog of White Collar-1 (WC-1) PhR/TF, first described in the non-pathogenic fungus *Neurospora crassa* [16,17]. The (blue) light-sensing ability of this protein resides in the light–oxygen–voltage (LOV) domain, which allows chromophore binding through flavin adenine dinucleotide. Through two additional protein–protein interaction domains termed Per-Arnt-Sim (PAS), WC-1 interacts with White Collar-2 TF, forming the white collar complex (WCC) [18]. Although the functioning of the *B. cinerea* WCC has been experimentally validated [13], including that (blue) light modulates BcWCL1-BcWCL2 protein–protein interaction dynamics [15], and that infection studies have confirmed smaller lesions produced by mutants lacking BcWCL1 in the presence of light [14], the extent of the light-triggered BcWCL1-dependent transcriptional responses during fungal infection remains unknown.

To this end, as the first approach to understanding the transcriptional impact of BcWCL1 on light-mediated responses, we determined genome-wide differentially expressed genes (DEG) using single and dual-organism RNA-seq approaches after 60 min of (white) light pulse (LP) in *A. thaliana*. For comparative purposes, we also determined DEG during saprophytic growth, referred to herein as non-infecting conditions, on cellophane-covered potato dextrose agar (PDA) plates, also employing a 60 min LP. 

## 2. Results

### 2.1. Light-Dependent Transcriptional Responses Seen in the ∆bcwcl1 Strain Indicate That This Mutant Is “Blind” Only during the Infection of Arabidopsis Leaves

**Figure 1:**
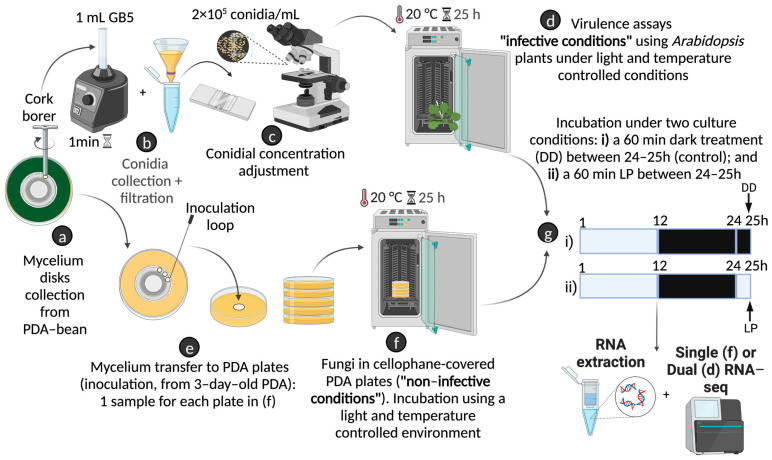
Schematic representation of the experimental setup used to determine genome-wide light-regulated transcriptional changes in *B. cinerea*. The top section of the figure denotes the virulence assays used to assess light-dependent *B. cinerea* transcriptional responses when infecting *A. thaliana*. The bottom section indicates the in vitro (non-infective) procedure, employing PDA Petri dishes for comparative purposes. First, (a) *B. cinerea* conidia were collected from a well-sporulated PDA-bean plate. Then, after conidia collection (b) and concentration adjustment (c), virulence assays (d) were performed in LD at 20 °C within temperature-controlled incubators (see Section 4). At the same time, *B. cinerea* inoculated on PDA plates (e) was transferred to LD culture conditions as denoted in (f). Finally, as indicated in (g), in both “infective” (d) and “non-infective” (f) conditions, a light pulse (LP) experiment was conducted. The rectangular boxes denote the 12 h:12 h photoperiod (white: light; black: darkness) and the 60 min dark (DD) and LP treatments used to determine genome-wide light-dependent transcriptional responses.

Light influences various biological processes in *B. cinerea*, including virulence and development (reviewed by [12]). Although early and late transcriptional responses to light have not been systematically studied in this organism [12,14], different lines of evidence indicate that significant changes in mRNA accumulation occur after a 60 min LP. Therefore, to determine how this ubiquitous and celestial cue modulates *B. cinerea* infection at the transcriptional level and the role of BcWCL1 TF/PhR in light-regulated transcriptional phenomena, we evaluated the effects of a 60 min LP during the infection of *A. thaliana* plants and under non-infective in vitro conditions (*B. cinerea* growing on PDA culture). During plant infection, the 60 min LP was provided 24 h after initiating the interaction (24 hpi). This condition mimics the onset of dawn, where exposure to light is expected to trigger important changes. Additionally, this time coincides with several events that occur during the development of the disease [7]. Finally, 60 min should allow the identification of “early light-responsive genes” whereas longer exposures (i.e., 120 min) would provide confounding results as “late light-responsive genes” may be already significantly expressed [19]. The experimental setup is illustrated in Figure 1.

Collectively, 1434 genes exhibited differential expression after the 60 min LP, regardless of the culture conditions and *B. cinerea* genotype under analysis (Table S1). Of note, the number of DEGs (up- and downregulated) upon the LP was significantly smaller during *Arabidopsis* infection (11.5% of the total DEG number in B05.10 and ∆*bcwcl1*) than in non-infective in vitro growth on PDA plates. Indeed, as depicted in Figure S1 and Table S1, the vast majority (80.8%) of DEGs were determined only during non-infecting conditions, indicating that *B. cinerea* light responses are decreased when the infection process is taking place. An even smaller number of genes were shared between both culture conditions (7.7%). The GO analysis did not reveal any enriched terms in the latter group. However, when infecting *Arabidopsis* leaves, the analysis revealed GO terms that were mostly related to carbohydrate metabolic processes. In contrast, GO enrichment analysis under non-infecting conditions revealed different processes associated with protein translation and energy, including ATP synthesis and the citric acid cycle (Figure S2). 

**Figure 2:**
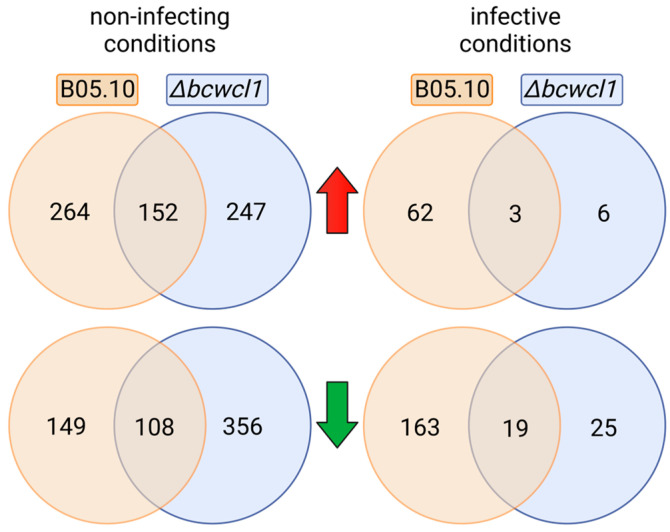
Venn diagrams of DEGs determined after a 60 min light pulse in *B. cinerea* under non-infective and infective conditions (left and right panels, respectively). For each culture condition, DEGs are shown as light-induced and light-repressed genes (LIG and LRG; red and green arrows, respectively). Exclusive DEGs are indicated in the outer circles, while the overlap, representing genes expressed in both genetic backgrounds, is shown in the inner circles.

The effect of the genotype on gene expression was also analyzed. The ∆*bcwcl1* mutant strain showed only nine light-induced genes (LIG) and 44 light-repressed genes (LRG) during *A. thaliana* infection (Figure 2, right panel). In contrast, when grown on PDA plates, the mutant showed 399 LIG and 464 LRG (Figure 2, left panel). Therefore, although there is a relevant number of BcWCL1-independent light responses in the mutant when examined in vitro, in planta, this number drops down, likely reflecting a massive remodeling of the underlying gene regulatory network in the latter condition. Interestingly, the number of LRGs observed for ∆*bcwcl1* during non-infecting conditions was more extensive than that of the B05.10 strain (464 and 257, respectively). In contrast, the B05.10 strain showed a more significant number of DEGs during *Arabidopsis* infection in comparison with ∆*bcwcl1* (four- and seven-fold increases; details in Table S2). In agreement with the latter observation, light-treated samples from the B05.10 strain infecting plants clustered more closely than any other cluster observed in the principal component analysis (PCA) (Figure S3), suggesting a particular transcriptomic state for these samples (see below). PCA also indicated that PC1 accounted for 71.15% of the observed total variation, segregating noninfective from infective culture conditions. The second component, explaining only 5.66% of the variance, segregated the culture conditions depending on the absence or presence of light.

**Figure 3:**
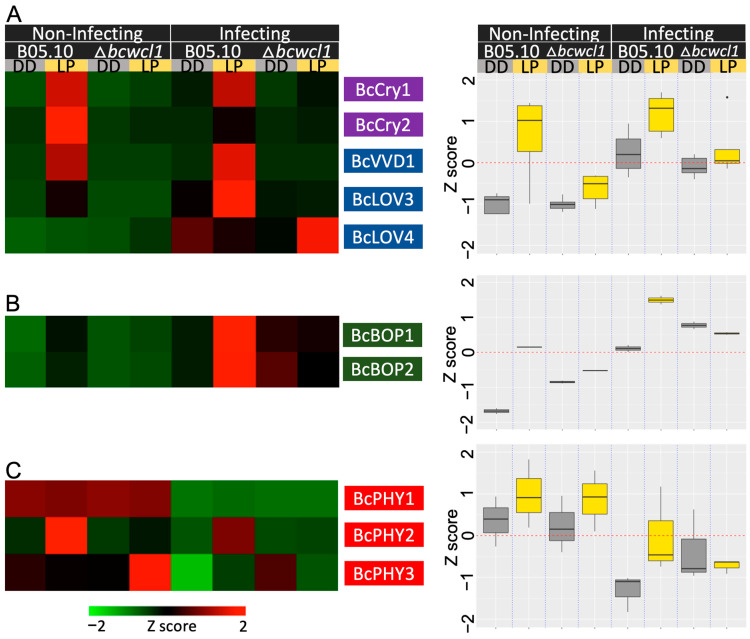
Clustering analysis of mRNA expression levels for all *B. cinerea* photoreceptor (PhR) encoding genes showing differential gene expression in response to a 60 min LP under non-infecting and infecting conditions. Heatmaps (left) and boxplots (right) illustrate normalized RNA-seq expression data, ranging from saturated green for log2 ratios −2.0 and below to saturated red for log2 ratios +2.0 and above. (**A**) Blue-light, (**B**) green-light, and (**C**) red-light receptors. A single row of colored boxes represents each PhRs. The average gene expression for each group of PhR is shown in the right panel.

Since the gene expression data show an important alteration in light transcriptional responses between the B05.10 strain and the ∆*bcwcl1* mutant, careful attention was placed on the expression of the genes encoding the other ten PhRs in *B. cinerea.* It has been previously reported that white light stimulation induces the transcript levels of these genes [12]. As shown in Table S3, while the B05.10 strain displayed seven light-induced PhRs genes during non-infecting conditions, three were still light-induced in the ∆*bcwcl1* mutant (*bccry1*, *bccry2*, and *bcphy2*). This indicates that under this condition, ∆*bcwcl1* behaves only as a “partially blind” mutant as previously suggested, and not as a fully blind fungus as expected for example from a *N. crassa* Δ*wc-1* strain [14]. In contrast, during the infection of *A. thaliana*, five PhRs genes out of 65 LIG in the B05.10 strain were upregulated by the LP, but none of them were in the ∆*bcwcl1* mutant. To gain deeper insight into this observation, and because *bcwcl1* was previously reported not to be light-induced [14], we analyzed the expression of the remaining PhR-encoding genes (Figure 3). Under non-infecting conditions, LP induced the expression of blue-light PhRs (Figure 3A, top), with *bclov4* being the only exception. In contrast, this light response was attenuated (in terms of fold change) during *A. thaliana* leaf infection. The overall expression levels of the green-light receptors *bcbop1* and *bcbop2* were higher during *A. thaliana* infection (Figure 3B), while the expression levels of the phytochromes were lower (Figure 3C). 

Taken together, the global gene expression analysis indicates that the ∆*bcwcl1* mutant (devoid of a single photoreceptor) can be considered as a facultative “blind” during the infection of *Arabidopsis* leaves, but not in vitro, where important light responses were reported [12].

### 2.2. The B. cinerea DEGs Determined during Non-Infecting Conditions Are Mainly Associated with a Reduction in Energy Production in Response to Light

**Figure 4:**
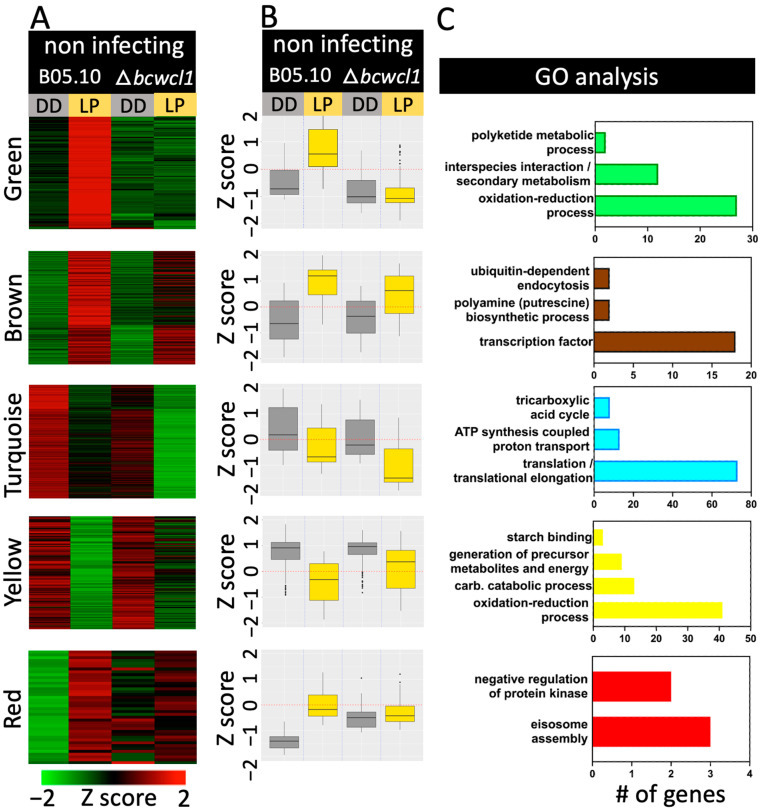
DEGs cluster analysis and overrepresented GO terms for genes during non-infecting conditions. The figure depicts the five major color-coded clusters of heatmaps (**A**) or boxplots (**B**) of co-regulated genes (from top to bottom, green, brown, turquoise, yellow, and red). Normalized RNA-seq expression data are indicated, ranging from saturated green for log2 ratios −2.0 and below to saturated red for log2 ratios +2.0 and above. Clustering analysis was performed using WGCNA. In (**A**), each gene is represented by a single row of colored lines. In (**B**), each boxplot represents the average expression of all genes contained in each cluster. In (**C**), the number of genes in different GO categories for biological processes is shown. Overrepresented categories are indicated compared to the entire set on the genome. The coloring of each bar matched those of each cluster, as indicated in (**A**). DD: darkness, LP: 60 min light pulse.

To determine expression trends, DEGs observed upon light stimulation under non-infecting conditions were subjected to a cluster analysis of gene expression levels. Five major color-coded clusters of coregulated genes are shown in Figure 4 (Table S4).

The green cluster displays BcWCL1-dependent LIGs (87 genes). The over-represented GO terms included several oxido-reduction biological functions and secondary metabolic processes. The light pulse led to the upregulation of seven different alcohol dehydrogenase-encoding genes, gene ID Bcin07g04460 encoding a sulfite oxidase (*bcsox1*), and genes such as *bchem15*, *bccox15*, and *bcgod1* (Bcin06g03980, Bcin16g00160, and Bcin14g05500, respectively) encoding a ferrochelatase required for the heme biosynthetic pathway and a conserved target of light regulation in fungi [20], a protein necessary for heme hydroxylation, and a glucose-oxidase-encoding gene (H_2_O_2_ production), respectively. Careful examination of the expression data also led to the identification of regulatory genes, including the light-responsive gene *bcfrq1*, which encodes the negative element of the *B. cinerea* circadian clock [9,14], and six genes encoding TFs, such as *bcltf3* (light transcription factor), which regulates conidiation [21]. Furthermore, 11 of 15 genes within the previously described botcinic acid biosynthesis gene cluster located in the subtelomeric region of chromosome 1 [22] were also significantly induced upon an LP dependent on BcWCL1. Since secondary metabolic processes were enriched in the GO analysis, we examined the expression patterns of all botcinic acid biosynthesis genes (Figure S4), showing higher expression levels during *Arabidopsis* infection and strong light inducibility during non-infecting conditions. Although some of the genes present in the cluster are induced during plant infection, the loss of botcinic acid production does not affect virulence [23]. 

In the brown cluster (Figure 4), several LIGs were determined in the B05.10 strain and the ∆*bcwcl1* mutant. This group of genes included PhRs in both genetic backgrounds (Table S3). Among the very few biological functions identified in the GO enrichment analysis, terms such as “polyamine/putrescine biosynthetic process” and “ubiquitin-dependent endocytosis” were determined. Polyamines, including putrescine, are a class of organic compounds that play essential roles in various physiological and developmental processes of plants, animals, and microorganisms. One of the primary functions of these polyamines is their ability to counteract multiple stressors, including reactive oxygen species, acting directly as scavengers as well as through the regulation of gene expression [24]. Moreover, 18 TF-encoding genes were differentially regulated by light in *B. cinerea.* These are described in Table S5. Among them, we observed the previously described *ltf1* [25], *bcwcl2* [13,14], and *bcatf1* [26]. 

The turquoise and yellow clusters, on the other hand, display light-repressed genes in the B05.10 and ∆*bcwcl1* strains. GO-enriched functions in the former cluster include translation/translational elongation and energy-related GO terms, such as ATP synthesis and the tricarboxylic acid cycle, suggesting the inhibition of energy production in response to light. In connection with this observation, the yellow cluster also contains energy-related GO terms among LRGs (Figure 4). Finally, with only 38 genes, the red cluster displays GO terms associated with the regulation of protein kinases and, interestingly, the GO term “eisosome assembly.” These molecular structures are lipid-protein microdomains in the plasma membrane, characterized by their rapid organization upon metabolic changes that disrupt proton gradients, among other stressors [27,28]. In the aggregate, in response to light, transcriptomic data indicate that the fungus displays extensive transcriptional changes involving several TFs, affecting several oxido-reduction biological functions including a reduction in energy (ATP) generation.

### 2.3. B. cinerea Reduces the Expression of Virulence Factors upon Illumination

**Figure 5:**
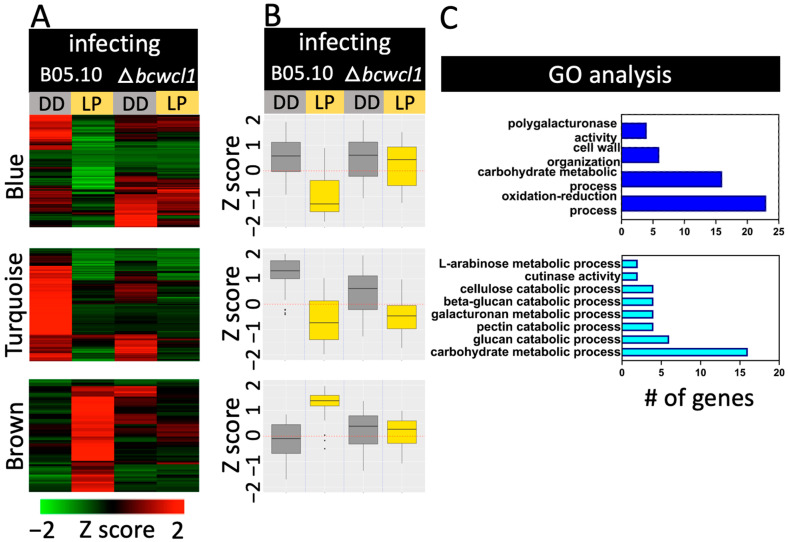
Cluster analysis of DEGs and overrepresented GO terms for *B. cinerea* during the infection of *A. thaliana* leaves. The figure depicts three color-coded clusters of heatmaps (**A**) or boxplots (**B**) of co-regulated genes (from top to bottom, blue, turquoise, and brown). Normalized RNA-seq expression data, ranging from saturated green for log2 ratios −2.0 and below to saturated red for log2 ratios +2.0 and above, is indicated. Clustering analysis was performed as indicated in Methods. In (**A**), each gene is represented by a single row of colored lines. In (**B**), each boxplot represents the average expression of all genes in each cluster. In (**C**), the number of genes in different GO categories for biological processes is shown. No GO categories were determined for the brown cluster. Overrepresented categories compared to the entire gene set on the *B. cinerea* genome are indicated. The coloring of each bar matched those of each cluster name, as shown in (**A**). DD: darkness, LP: 60 min light pulse.

In contrast to the abovementioned remarks, during the infection of *Arabidopsis* leaves, we determined a lower *B. cinerea* transcriptional response to LP, with only 276 DEGs in both genotypes (223 only in B05.10, 29 only in ∆*bcwcl1*, and 24 in both strains). Only three groups of co-regulated genes in *B. cinerea* were observed (Table S6). The blue cluster in Figure 5 displays BcWCL1-dependent LRGs. GO terms were mainly represented by carbohydrate processes and stress-related functions. Importantly, this cluster also included biological and molecular functions related to the degradation of the plant cell wall, represented by *bcpme1*, *bcpgx1*, *bcpg1/3/4*, Bcin08g02390 encoding a peptidase, and virulence-related genes, including *bcboa17*, *bcpme1*, and *bcnep2* (Table S6). The turquoise cluster also displayed LRGs; however, with the presence of BcWCL1-dependent and-independent genes (Figure 5, Table S6). In this latter group of genes, we observed different plant-cell-wall-related biological and molecular functions, including those of pectin esterases, proteases, and cutinases. In both the blue and turquoise clusters, several light-repressed glycosyl hydrolase (GH)-encoding genes (14 and 22, respectively) representing 16 different GH families were identified (Table S7). In contrast, the brown cluster is the only set of *B. cinerea* genes displaying light inducibility during the infection of *Arabidopsis* leaves, but no enriched GO terms were identified. Genes include the light-induced and WCL1-dependent *bcfrq1* [9,14] and the recently characterized gene *bcfet1* (formerly and incorrectly termed *bclcc13*) encoding a ferroxidase (and not a laccase) of the reductive iron assimilation system in which absent, hypervirulent, or increased infection is observed [29]. Additional genes include *bcboa8* [22], *bcltf1* [25], and those of photoreceptors (Table S3). 

### 2.4. Arabidopsis Global Gene Expression Correlates with the Downregulation of the Virulence Factors of B. cinerea upon Illumination

**Figure 6:**
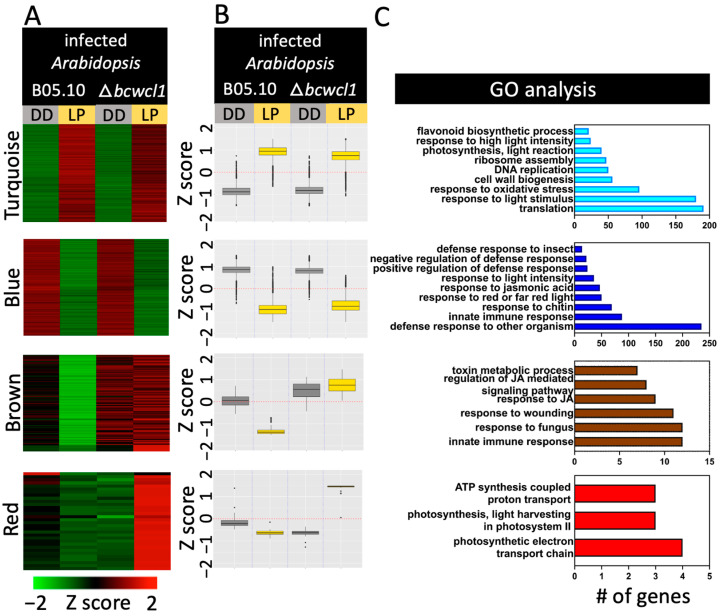
Clustering analysis of DEGs identified in *A. thaliana* infected by *B. cinerea*. The figure depicts four major color-coded clusters of heatmaps (**A**) or boxplots (**B**) of co-regulated genes (from top to bottom, turquoise, blue, brown, and red). Two additional clusters of co-regulated genes are indicated in Figure S7. Normalized RNA-seq expression data, ranging from saturated green for log2 ratios −2.0 and below to saturated red for log2 ratios +2.0 and above, is indicated. Clustering analysis was performed as shown in Methods, using WGCNA. In (**A**), each *Arabidopsis* gene is represented by a single row of colored lines. In (**B**), each boxplot represents the average expression of all genes contained in each cluster. In (**C**), the number of genes in different GO categories for biological processes is shown. Overrepresented categories compared to the entire set on the *A. thaliana* genome are indicated. The coloring of each bar matched those of each cluster name, as indicated in (**A**). DD: darkness, LP: 60 min light pulse.

Finally, using the pathogen:host RNA-seq strategy depicted in Figure 1, we also determined the major light-dependent transcriptional changes occurring in *A. thaliana* when infected with *B. cinerea.* Regardless of the fungal genotype, 8905 genes were considered differentially expressed upon light stimulation (Table S8), with 61.6% of them differentially expressed upon challenge with B05.10 and *∆bcwcl1* (Figure S5). The weighted correlation network analysis (WGCNA) revealed four major clusters of coregulated genes (Figure 6, Table S9). Irrespective of the fungal strain with which *Arabidopsis* was infected, the turquoise and blue clusters displayed LIG and LRG. The first cluster contained GO terms related to LP, such as photosynthesis, oxidative response, and flavonoid biosynthesis. The blue cluster of LRGs also contained a group of transcripts associated with light terms, including “response to red or far-red light”. Interestingly, and consistent with the major findings depicted in Figure 5 (see above), different GO terms related to plant defense responses were enriched among those genes repressed upon the LP, including “response to chitin” an abundant polysaccharide that provides strength to the cell walls of fungi. In agreement with these observations, the brown cluster of Figure 6 also displayed GO terms that include several defense-related entries, such as the defense response to fungi, the regulation of jasmonic acid, and the “response to jasmonic acid,” an important phytohormone that orchestrates plant defense responses to necrotrophs. In the red cluster, only energy and photosynthesis-related GO terms were identified among DEGs. This particular group of *Arabidopsis* genes was highly induced upon light stimulation, but only during the infection with the ∆*bcwcl1* mutant. Two other clusters of coregulated genes were identified (Figure S7; Table S9), but no enriched GO terms were determined.

## 3. Discussion

The responses of *B. cinerea* to light are complex because they not only regulate developmental processes, such as conidiation and sexual differentiation, but also play a crucial role in *Botrytis’* interactions with other organisms, such as plants [14,30] or fungi [31]. Therefore, in this study, we aimed to explore the global transcriptional impact of BcWCL1 on light-mediated responses, not just in classic lab settings (PDA Petri plate), but also in conditions where relevant biotic stimuli (plant interaction) are present. Half a century ago, a series of investigations revealed that *B. cinerea* can sense light and respond to it with morphological changes that depend on the quality of the light applied to the fungus [32,33,34,35,36]. The light spectra included near-ultraviolet (NUV), blue, red, and far-red lights. To accommodate this spectral sensitivity range, the fungus possesses 11 photoreceptors encoded in its genome [12]. Although deleting a single blue light perception protein such as BcWCL1 [15] is unlikely to render the mutant fully “blind”, previous studies on the *∆bcwcl1* mutant have shown that it is only partially deficient in its response to (white) light [11,14]. Thus, several BcWCL1-dependent light responses have been observed for homologs of well-known light-responsive genes in fungal systems, including *bcfrq1*, *bcvvd1*, and *bccsp1* [37], but some TF-encoding genes respond to light independently of BcWCL1. However, in these experiments, only the expression of a few genes was assessed [14]; therefore, the extent of this observation was inconclusive. Different genome-wide transcriptional behaviors have been reported in other filamentous fungi, such as *N. crassa* and *Trichoderma atroviride*, where, in the absence of the WC-1 orthologue, responses to light are nearly entirely lost [19,38]. However, in *Aspergillus nidulans*, transcriptional responses to light appear more complex, as observed in *B. cinerea* [39]. So far, information regarding light-triggered genome-wide transcriptional responses in *B. cinerea* is limited [25,40], and the role of light in the infection process remains largely unexplored. To understand how light orchestrates transcriptional responses in *B. cinerea*, mutant strains of light transcription factors (termed LTFs) have been generated [14,21,25,41]. The virulence of these mutants has been assessed, showing that only ∆*bcwcl1* and ∆*bcltf1* display a decreased ability to infect plants. In a study published by Schumacher et al. [25], the transcriptomic response of BcLTF1 was evaluated after a 60 min LP culture on Petri dishes, which induced the light-dependent expression of 244 genes, with 66.8% of them being BcLTF1-dependent. Similar results were observed for BcWCL1, with 63.4% of the 416 light-induced genes in the B05.10 strain being BcWCL1-dependent. However, differences were noted in the light responses of the two mutants, such as the light induction of some PhRs encoding genes (in particular, *bcvvd1*, *bclov3*, *bcbop1*, *bcbop2*, and *bcphy2*), which was dependent on BcWCL1 but not on BcLTF1. This suggests that other TFs and additional signaling pathways may directly control the expression of a significant number of light-regulated genes, including BcLTF2, which was found to have a greater regulatory potential for light-regulated genes based on gene regulatory network analyses [40]. While all these studies provide great insights into the photobiology of *B. cinerea*, a major caveat is that they may not fully reflect key aspects of the fungus biology. Indeed, all light transcriptomic analyses have been conducted in axenic conditions, as the fungus grows in culture media. Such a setting is simpler compared to an infection paradigm. Indeed, assays in planta include major biotic cues provided by the host, which can largely remodel the regulatory landscape and lead to a completely different response upon additional cues such as light. In this regard, it is not entirely clear to what extent gene expression changes were due to the presence of plants in infectious conditions or simply to the presence of plant tissue and its components. Therefore, it might be interesting to analyze in the future global gene expression patterns upon light stimulation under infection conditions as performed herein and in Petri dishes containing media supplemented with the same plant tissue or different fractions, to be able to unravel subtle variations observed in response to light. By conducting such experiments, we may obtain valuable insights into the contribution of different plant components and their roles in modulating gene expression patterns during infection. Moreover, this could help us disentangle the effects of plant-derived signals and those directly induced by the pathogen, providing a better understanding of the underlying molecular mechanisms.

Herein, on the one hand, *B. cinerea* was grown in vitro conditions and subjected to a 60 min white LP; the number of DEGs was significantly affected, where we could distinguish BcWCL1-dependent and independent groups of transcripts. Numerous TFs induced by light, exceeding the number of previously defined LTFs [12] (for example, Bcin01g08080, Bcin03g03310, Bcin04g01840, Bcin06g03510, Bcin07g02960, Bcin08g02020, Bcin10g03530, Bcin11g00730, Bcin16g01770, and Bcin16g03230), should amplify or modify the light-dependent transcriptional response. However, this was not observed when the fungus infected *A. thaliana*, at least under culture conditions and the timing of infection reported herein. Only the B05.10 strain, not the *∆bcwcl1* mutant, showed a significant number of LIGs and LRGs in the virulence assays. In this case, circa 10% of the LIGs in the B05.10 strain on *Arabidopsis* were PhRs (5 out of 65 LIGs). Despite this observation, the range of the transcriptional induction of genes was rather small when infecting *Arabidopsis*, which is the opposite of what has been described in the vast majority of fungal systems described as photobiological models [37,42], where photoreception mainly induces genome-wide transcriptional changes (employing in vitro conditions). Therefore, the results presented herein indicate that in *B. cinerea* light signals result in broad and narrow responses (in terms of the number of genes displaying differential expression) that affect transcript accumulation, which seem highly dependent on culture conditions, particularly when the latter include complex sets of biotic signals. 

The overall expression patterns of *B. cinerea* genes encoding green- and red-light PhRs during the infection of *A. thaliana* leaves deserve further attention. We observed higher mRNA levels for *bcbop1* and *bcbop2*, which encode the green light receptor opsin and lower expression levels for the three phytochromes. It is important to note that the leaves of plants with high chlorophyll and other photosynthetic pigments predominantly reflect green light [43], which could explain the higher expression levels of opsin-encoding genes. The biological significance of this observation is still speculative, but it is generally accepted that light serves as a signal of whereabouts in fungal systems [44]. Further research is needed to understand this group of light-sensing proteins reported to also behave as proton pumps. Although *bcbop1* in *B. cinerea* is dispensable for virulence [45], little is known about *bcbop2*. BcBOP2 is similar to the CarO opsin of *Fusarium fujikuroi* [46], which delays spore germination in this pathogenic fungus. Whether BcBOP2 signals in *B. cinerea* an appropriate surface for conidial germination remains to be investigated. In addition to green light, the plant canopy is characterized by strong red-light absorption necessary for photosynthesis, behaving as a mirror of large quantities of far-red light [43]. This far-red-shifted environment, the relevance of which to phytopathogens has not been analyzed in detail [30], may be related to the lower expression levels observed for *B. cinerea* phytochromes. Plants utilize the red:far-red ratio as a positive phototropic signal to grow towards light [43]. Although limited information is available on the phototrophic responses in *B. cinerea* (reviewed in [12]), the fungus displays clear conidial germ tube phototropism: positive towards red light and negative to NUV and blue light [47]. Shorter germ tubes, such as those obtained under NUV or blue light, are compatible with the formation of infectious structures. In *B. cinerea*, germ tube length is maximal under red light and is significantly shorter in the presence of far-red light, a quality of light that is only surpassed by NUV and blue light in terms of their effect on germ tube elongation [47]. Therefore, the lower mRNA levels of *B. cinerea* phytochromes observed on the leaf surface were compatible with the infection process, as reduced red light signaling was expected. Unfortunately, Islam et al. [47] did not assess combined wavelengths on *B. cinerea* germ tube elongation (e.g., green plus far-red light), which would better reflect the quality of light that the fungus faces on leaf surfaces. Yet, these reported observations and our new datasets raise interesting hypotheses to be tested in planta, to better integrate *B. cinerea* gene expression datasets.

Compared to constant dark conditions, the presence of constant light or photoperiod during the interaction between *B. cinerea* and *A. thaliana* results in smaller necrotic lesions, as observed in previous virulence assays [14]. In addition, BcWCL1 is required for full virulence when exposed to constant light and LD, as previously reported [14]. So, why are smaller lesions obtained when *Arabidopsis* faces the *∆bcwcl1* mutant? This outcome may result from at least two possible scenarios: the first scenario is that *Arabidopsis* displays an increased transcriptional defense response when interacting with the *∆bcwcl1* mutant compared to B05.10. However, this is unlikely because most *Arabidopsis* genes were either induced or repressed by the LP, regardless of the *B. cinerea* strain used in the virulence assays. Indeed, the two major clusters of co-regulated *Arabidopsis* genes (turquoise and blue in Figure 6) account for over 96% of DEGs. Indeed, as expected after a short LP, an important group of LIGs in *Arabidopsis* exhibit biological functions associated with photosynthesis and responses to light stimulus. Nevertheless, the brown cluster depicted in the same figure provides an alternative explanation, as *Arabidopsis* LRGs associated with defense functions in plants were downregulated when infected with B05.10, but not with ∆*bcwcl1*. The second scenario is that the *∆bcwcl1* mutant displays a reduced expression of virulence genes, which is also unlikely since the LP downregulates the expression of virulence-associated genes in both B05.10 and *∆bcwcl1.* Indeed, the turquoise cluster, the largest group of genes in Figure 5, represents 39% of DEGs. Consequently, in a BcWCL1-dependent or independent manner, LRGs, including polygalacturonases such as BcPG1, which is required for full virulence in *B. cinerea* [48], glycosyl hydrolases, pectin esterases, proteases, and cutinases, were determined. This reflects the broad genetic repertoire of fungal virulence factors present in *B. cinerea* [1,7]. Although a combination of the two aforementioned scenarios is also possible, there were no light-induced PhRs in the *∆bcwcl1* mutant during the *Arabidopsis* infection. Thus, the lack of blue light signaling in the ∆*bcwcl1* genetic background could lead to longer germ tubes as explained earlier [47], or more elongated non-penetrating hyphae, delaying or hampering the infection. 

Overall, the patterns of expression of virulence factors and DEGs in both the plant and fungus showed a complex cross-talk between infection and defense-associated biological functions regulated by light. Collectively, upon illumination at 24 hpi in *Arabidopsis*, the fungus seemed to arrest infection, which could lead to a decrease in the transcriptional defense response of the plant. This observation is consistent with a previous halt in *B. cinerea* growth observed in *Arabidopsis* between 20 and 28 hpi [49]. For plants, this may reflect the well-studied growth and defense trade-offs [50]. Consistent with the aforementioned lack of blue light signaling and the arrest of infection upon illumination, *bcfrq1*, encoding the negative element of the *B. cinerea* circadian clock [9,14], was induced upon light exposure when infecting *A. thaliana* only in the B05.10 strain. Interestingly, the overexpression of *bcfrq1* in *B. cinerea* leads to a reduction in the lesion size observed in *Arabidopsis* leaves, while its loss-of-function mutant (∆*bcfrq1*) displays increased virulence [9]. Thus, the LRGs in *B. cinerea* determined during *Arabidopsis* infection are strong candidates for circadian regulation and could partly explain the time-of-infection-dependent outcome demonstrated in the *B. cinerea*–*A. thaliana* pathosystem [9]. High-resolution time-course circadian experiments employing a dual pathogen:host RNA-seq strategy are required to provide definitive conclusions. 

## 4. Materials and Methods

### 4.1. Botrytis Cinerea Strains and Regular Culture Conditions Employed for Propagation

*Botrytis cinerea* Pers. Fr. (*Botryotinia fuckeliana* [de Bary] Whetzel) strain B05.10, originally isolated from a *Vitis vinifera* vineyard in Germany, was used as the wild-type strain for the experiments. In addition, the same and previously generated *B. cinerea* knockout mutant of BcWCL1 (hereinafter referred to as ∆*bcwcl1*) [14] was used in the present study. B05.10 wild-type and ∆*bcwcl1* strains were cultivated and kept on Petri plates containing potato dextrose agar PDA (Becton, Dickinson, Franklin Lakes, NJ, USA) supplemented with mashed primary bean leaves (50 g/L; PDA-bean) at 20 °C.

### 4.2. Culture Conditions Employed for LP Experiments

Two 60 min light pulses (LP) experiments were conducted to analyze the transcriptional impact of light on *B. cinerea* biology. In both cases, we used genome-wide mRNA sequencing (RNA-seq). The first set of LP experiments was conducted during the infection of *Arabidopsis thaliana* leaves, while the second set of experiments was performed under non-infective (in vitro) conditions (see below). 

First, we determined the effect of a 60 min LP on the transcriptome of *B. cinerea*, but during the initial stages of the infection of *A. thaliana* plants. Therefore, in this study, we performed RNA-seq of fungus and host during infection to determine the changes in gene expression in both organisms. Virulence assays were conducted as previously described [29]. For this procedure, *A. thaliana* plants were first propagated in a 12 h:12 h LD regime at 20 °C using Percival incubators (Percival Scientific, IA, USA) equipped with cool white-light fluorescent tubes (light intensity up to 100 µmol/m^2^/s; wavelength 400–720 nm) employing the commercial substrate green power complete mix (TopCrop, Valencia, Spain). For virulence assays, *B. cinerea* conidia were collected from a well-sporulated PDA bean plate (seven days old; Figure 1a). A final concentration of 2 × 10^5^ conidia/mL (in 7 µL) was suspended on Gamborg B5-2% glucose medium (GB5) with 10 mM KH_2_PO_4_-K_2_HPO_4_ at pH 6.4 (Figure 1b,c). Five-week-old *A. thaliana* (Col-0) plants were inoculated with the mentioned conidia suspension inside plastic boxes at 20 °C under a humid environment (Figure 1d). As mentioned above, two different lighting conditions were applied: the first set of infected plants (with B05.10 or ∆*bcwcl1 B. cinerea* strains) were incubated in LD for 24 h. After this period, between hours 24 and 25, this set of infected plants was kept in DD for 1 h and collected in the absence of light. The second set of infected plants was incubated for 60 min in light (LP) and then collected in the presence of light (Figure 1g). In the DD (control experiments) and LP treatments, infected leaves were flash-frozen and kept at −80 °C until RNA extraction. 

For comparative purposes, B05.10 and ∆*bcwcl1* strains were cultivated in Petri dishes containing PDA for three days at 20 °C, achieving regular mycelia growth. For this purpose, the same LD regimen used for *A. thaliana* was employed for *B. cinerea*. These plates were used as inoculum sources for the agar plugs, which is a routine procedure for *B. cinerea* (Figure 1e). After this period, agar plugs of both strains were inoculated in cellophane-covered PDA-containing Petri dishes in LD culture conditions for 24 h (Figure 1f). Between hours 24 and 25, one set of cultures was maintained and harvested under DD in a light-proof, temperature-controlled dark room, whereas the second set of plates was subjected to 60 min LP and collected in the presence of light (Figure 1g). All samples were kept at −80 °C until further purification. 

### 4.3. Total RNA Extraction and mRNA Sequencing

For total RNA extraction, 10 mg of frozen mycelia or the plant-mycelia mixed tissue (from virulence assays, see above, Figure 1) were ground to a powder using an automated tissue homogenizer (TissueLyser II, Qiagen, Hilden, Germany). In both cases, the TRIzol reagent (Invitrogen, Waltham, MA, USA) was used according to the manufacturer’s instructions. To measure RNA quantity and quality, a NanoDrop spectrophotometer (Thermo Scientific, Waltham, MA, USA) and a fluorescence-based capillary electrophoresis machine (Fragment Analyzer, Agilent, Santa Clara, CA, USA) were used. All samples displayed RNA integrity numbers (RIN) higher than 7.0. After high-quality RNA extraction, poly-A-containing mRNA molecules were obtained from the total RNA. Illumina libraries were constructed according to the manufacturer’s instructions using the TruSeq Stranded RNA Sample Preparation Kit (Cat no. 20020595, Illumina, San Diego, CA, USA). Fluorescence-based capillary electrophoresis was used to assess library integrity and size. Library sequencing was performed using the HiSeq2500 sequencer. The sequences of two independent biological replicates for each condition were obtained (single-end and paired-end sequences for the non-infected and infected conditions, respectively). In the pathogen:host RNA-seq experiment, since we analyzed the effect of a 60 min LP 24 h after the infection was initiated, the expected amount of fungal tissue was considerably less than that of leaves, which could have prevented the capture of fungal reads. Therefore, sequencing depth was increased to guarantee an adequate estimation of the DEGs (see below) in *B. cinerea* during the infection of *Arabidopsis* (particularly relevant for low-expressed genes). Thus, an average of 37.4 million reads were obtained for the dual pathogen:host RNA-seq experiment, which is considerably higher than the 9.98 million reads obtained for the standard (pathogen only) RNA-seq experiment.

### 4.4. Identification of Differentially Expressed Genes

FASTQC software (https://www.bioinformatics.babraham.ac.uk/projects/fastqc/, accessed on 9 May 2023; version 0.11.8) was used to assess the quality of the RAW sequence data. Trimmomatic software (version 0.39; critical parameters: SlidingWindow: 4:10; Leading: 10; Trailing: 10; Minlen: 25) was used to filter out sequencing adapters and low-quality reads [51]. Read mapping and counting were performed using the STAR software (version 2.7.9a) [52] against the *B. cinerea* reference genome retrieved from Ensembl Fungi [53]. This database was based on a previously published work [54]. To identify DEGs after LP during *A. thaliana* infection, reads were mapped and quantified using STAR against a combined in silico-constructed reference genome of both *B. cinerea* and *A. thaliana* transcriptomes, as described previously [55]. This simple procedure ensures the accurate mapping to each genome. DEGs between the control (DD) and treatment (LP) in each condition were determined using DESeq2 [56], which uses the Benjamini–Hochberg [57] method to adjust the *p*-values. DEGs were filtered based on an adjusted *p*-value < 0.05, with a log2 (fold-change) >0.5.

### 4.5. Clustering of Differentially Expressed Genes and Gene Ontology Analysis

DEGs were clustered on the R platform using the WGCNA library [58]. Initially, the getVarianceStabilizedData function was employed with the DESeq2-normalized counts to normalize the data with respect to the library size, obtaining a homoscedastic value matrix. Subsequently, clusters were obtained using the blockwiseModules function (critical parameters: power = 9, maxBlockSize = 10,000, minModuleSize = 10, TOMType = “signed”, corType = ”bicor”, deepSplit = 2, mergeCutHeight = 0.25, networkType = “signed hybrid”, maxPOutliers = 0.1, pearsonFallback = “individual”). The p.heatmap and ggplot2 data visualization packages for R were used to obtain Z-score heatmaps and graphs for clustered DEGs, respectively. For *B. cinerea*, the FungiFun2 [59] online platform was employed to evaluate gene ontology (GO) term enrichment among the clustered DEGs. In addition, we used the generic GO TermFinder open-source software (https://go.princeton.edu/cgi-bin/GOTermFinder; accessed on 9 May 2023) [60], employing a custom-made GO database for *B. cinerea* constructed using BLAST2GO [61]. For *A. thaliana*, the GO term enrichment tool available at The *Arabidopsis* Information Resource was used [62]. For all GO term analyses, a false discovery rate correction (*p* < 0.05) was applied to the over-represented GO terms after performing a hypergeometric test.

### 4.6. RNA-Seq Data Accession Numbers

The RAW data from the experiments performed in this study were deposited with the accession number PRJNA932210 in the NCBI SRA database. 

## Data Availability

The data supporting the reported results can be found in the NCBI SRA database under accession number PRJNA932210.

## Supplementary Materials

The supporting information can be downloaded at: https://www.mdpi.com/article/10.3390/ijms24108705/s1.
