# Supplementary material for: Genome-Wide Characterization of Light-Regulated Gene Expression in Botrytis cinerea Reveals Underlying Complex Photobiology
Source: Int J Mol Sci. 2023 May 13;24(10):8705. doi: 10.3390/ijms24108705 (PMC10218500; doi:10.3390/ijms24108705)
Supplement: Supplementary file 1 [file ijms-24-08705-s001.zip › Supplementary figures.pdf]

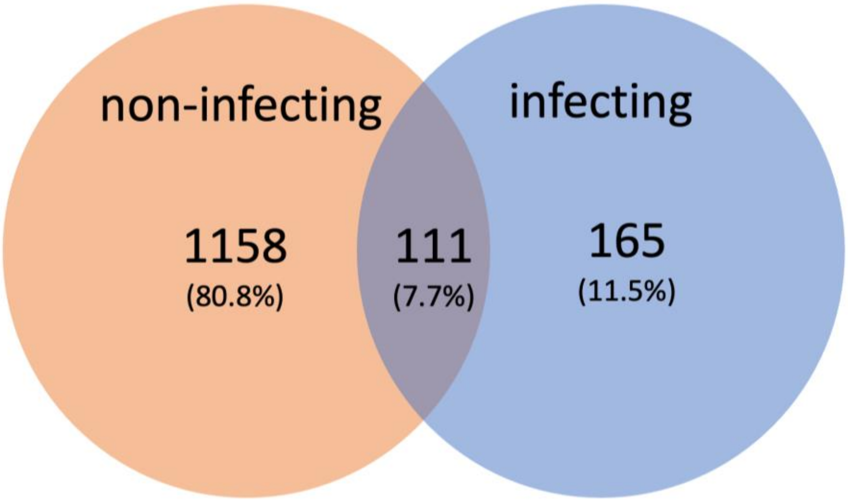

Figure S1: Venn diagram of DEG in *B. cinerea* upon light stimulation. The total number of light-responsive genes (up- and downregulated) was determined in *B. cinerea* (B05.10 and Δ*bcwcl1*) under non-infecting conditions and during the infection of *A. thaliana*. Transcript levels with a log2 fold-change ≥ 0.5 ($p < 0.05$) after the 60 min LP were considered DEGs.

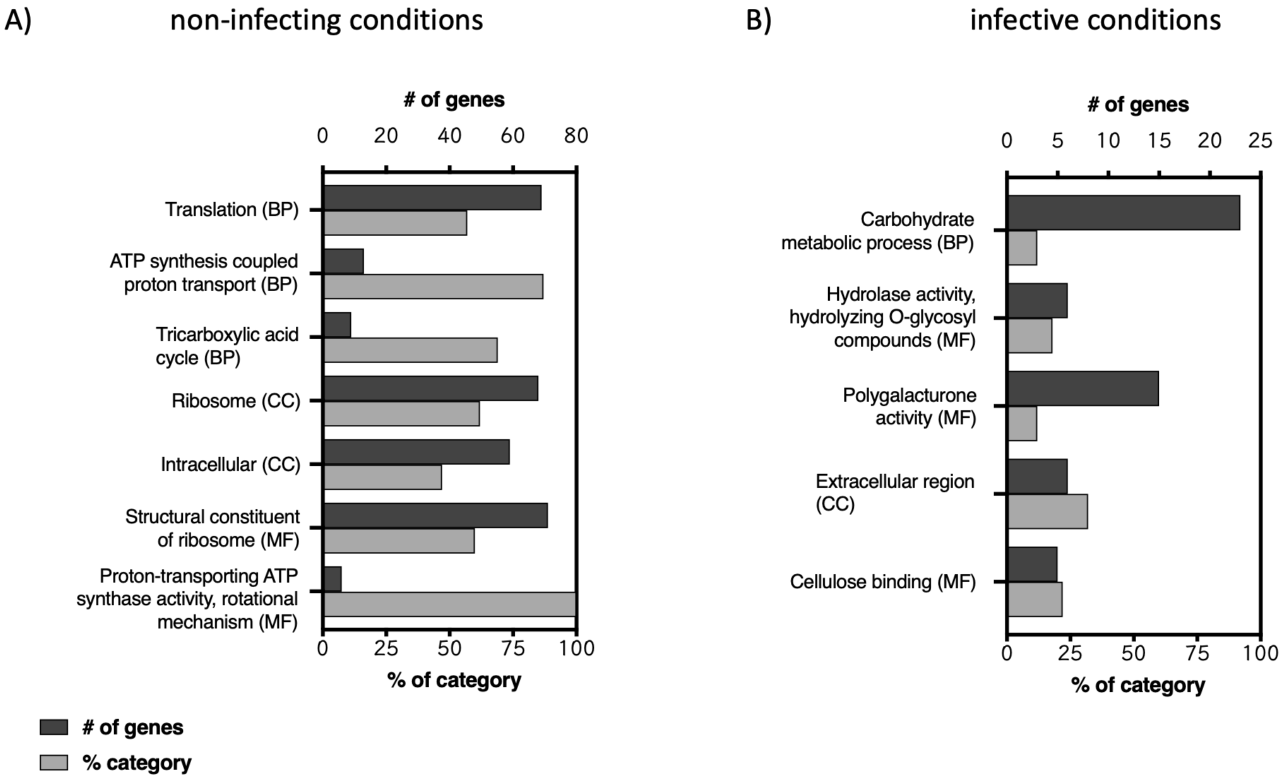

Figure S2: Overrepresented GO terms determined after enrichment analysis among *B. cinerea* DEGs observed after the 60 min LP during non-infecting (**A**) and infecting (**B**) conditions. The bars represent the number of genes observed in each category and their respective percentage (black and grey bars, respectively; BP, biological process; CC, cellular component; MF, molecular function).

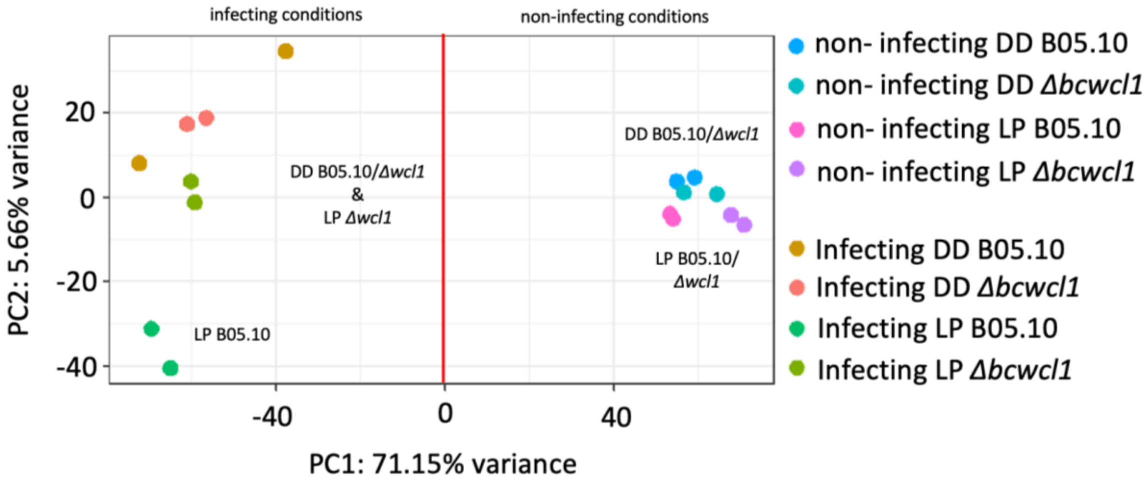

Figure S3: Principal component analysis (PCA) of normalized global gene expression data of *B. cinerea* after a 60 min LP during non-infecting conditions (right) and the infection of *A. thaliana* leaves (left). The PCA shows a particular transcriptomic state for the B05.10 strain after the LP during the infection of *A. thaliana*. Colored dots denote each biological replicate. "DD" and "LP" indicate darkness- and light-pulse-treated samples, respectively.

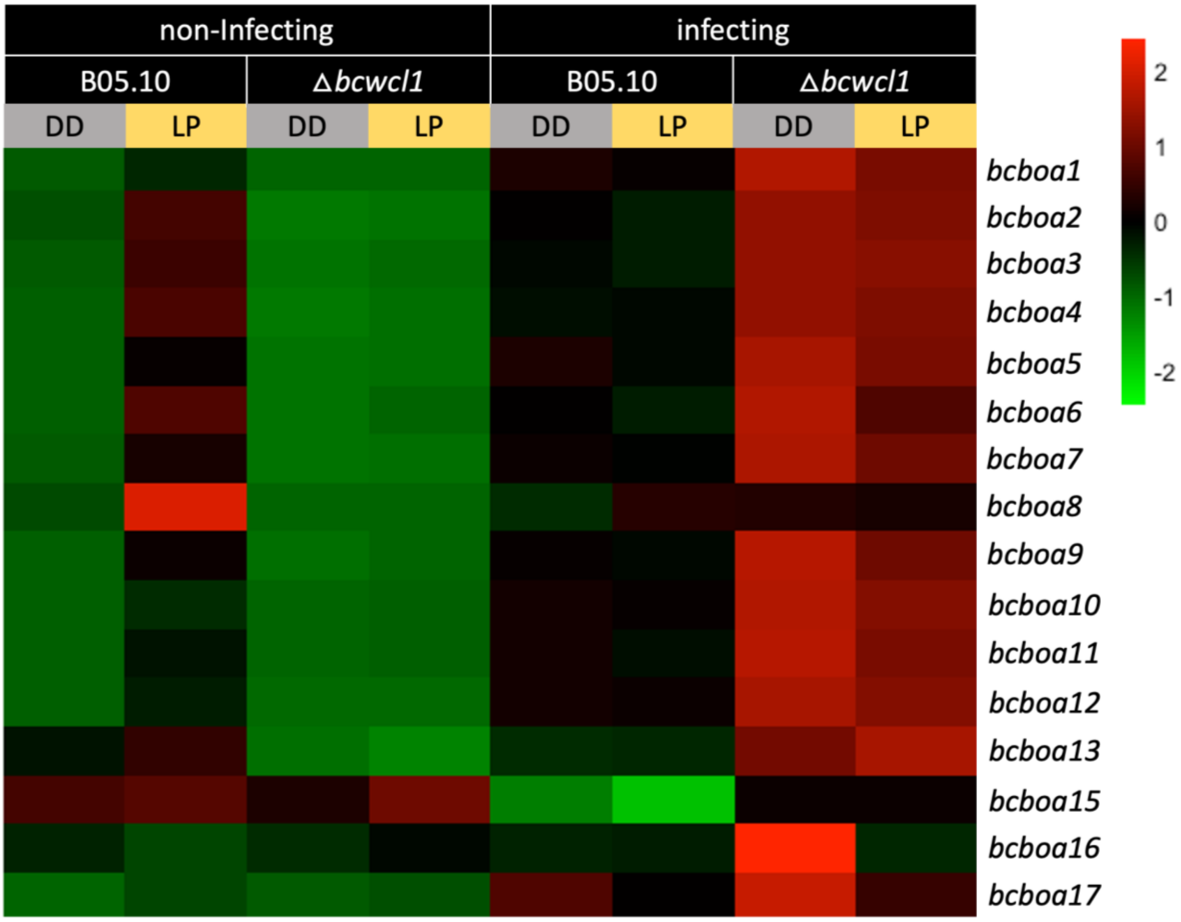

Figure S4: mRNA expression levels of the botcinic acid genes. The gene expression levels in response to a 60 min LP under non-infecting and infecting conditions (left and right panels, respectively) for B05.10 and Δ*bcwcl1* are shown. The heatmap represents normalized RNA-seq expression data, ranging from saturated green for log2 ratios −2.0 and below to saturated red for log2 ratios +2.0 and above. "DD" and "LP" indicate darkness and light pulse treated samples, respectively.

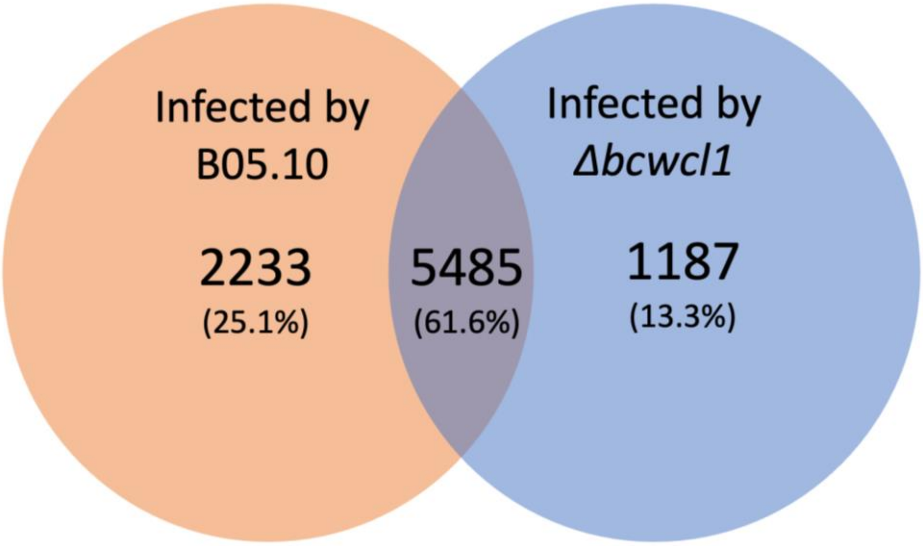

Figure S5: Venn diagram of differentially expressed genes in *A. thaliana* infected by *B. cinerea* B05.10 or *Δbcwcl1*. The total number of light-responsive genes (up- and downregulated) was determined in *A. thaliana* under infecting conditions. Transcript levels with a log2 fold-change ≥ 0.5 ($p < 0.05$) after the 60 min LP were considered DEGs.

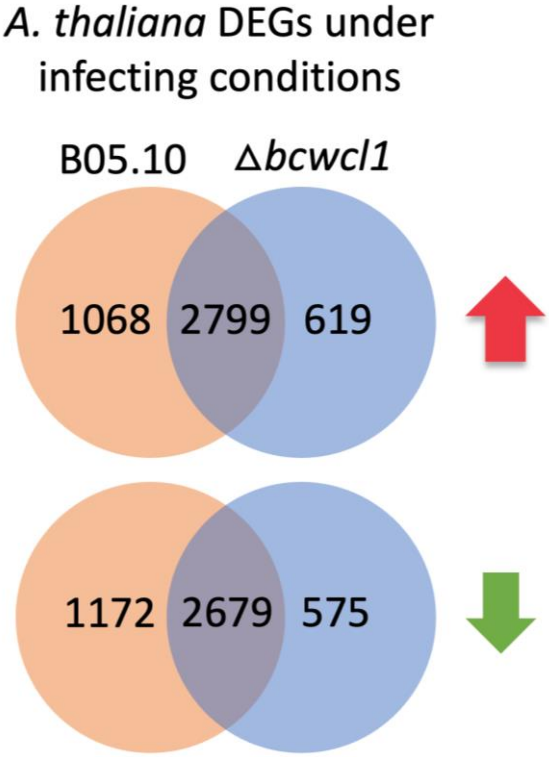

Figure S6: Venn diagrams of DEGs determined in *A. thaliana* infected by *B. cinerea* B05.10 or *Δbcwcl1*. The number of DEGs is presented as LIGs and LRGs (red and green arrows, respectively). For each condition, exclusive DEGs are evidenced in the outer circles, while the overlap representing DEGs in both genetic backgrounds is indicated in the inner circles.

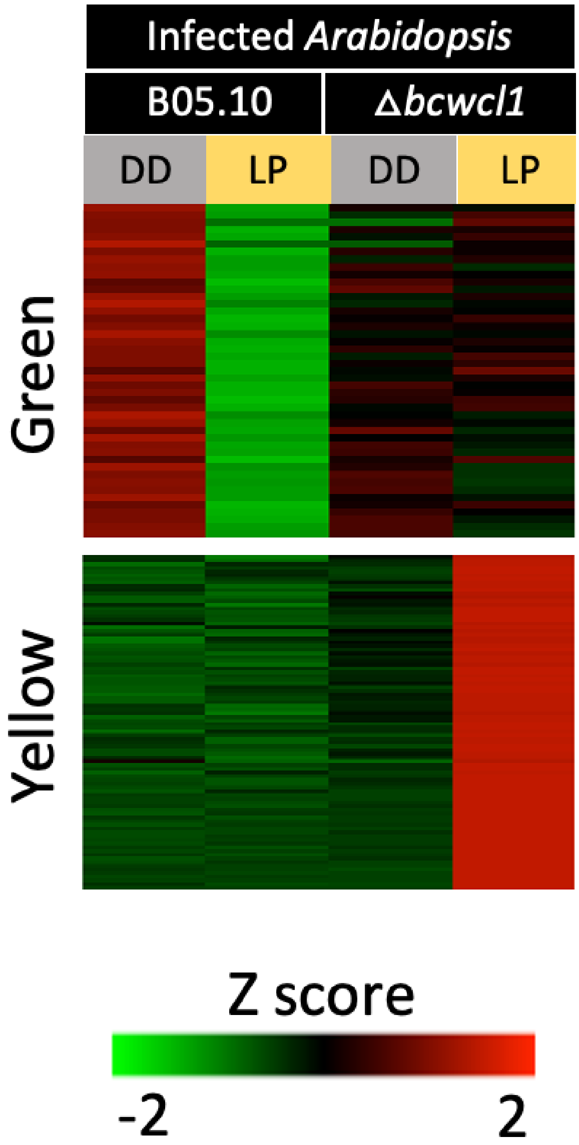

Figure S7: Clustering analysis of DEGs identified in *A. thaliana* during the infection of *B. cinerea*. The figure depicts two color-coded clusters of heatmaps of co-regulated genes (from top to bottom, green and yellow). Normalized RNA-seq expression data, ranging from saturated green for log2 ratios −2.0 and below to saturated red for log2 ratios +2.0 and above is indicated. A single row of colored lines represents each *Arabidopsis* gene. No enriched GO categories were determined. DD: darkness, LP: 60 min light pulse
